# High Dietary Inclusion of Faba Bean Improved the Meat Quality of Pacific White Shrimp, *Litopenaeus vannamei*, Rather Than the Growth Performance

**DOI:** 10.1155/anu/2534380

**Published:** 2025-12-09

**Authors:** Zhengri Gan, Yuting Xu, Xinyi Fei, Xiaoqin Li, Xiangjun Leng

**Affiliations:** ^1^ National Demonstration Center for Experimental Fisheries Science Education, Shanghai Ocean University, Shanghai, 201306, China, shou.edu.cn; ^2^ Centre for Research on Environmental Ecology and Fish Nutrition (CREEFN) of the Ministry of Agriculture and Rural Affairs, Shanghai Ocean University, Shanghai, 201306, China, shou.edu.cn; ^3^ Shanghai Collaborative Innovation for Aquatic Animal Genetics and Breeding, Shanghai Ocean University, Shanghai, 201306, China, shou.edu.cn

**Keywords:** faba bean, growth performance, intestinal microbiota, muscle quality, Pacific white shrimp, transcriptome

## Abstract

The study investigated the effect of replacing soybean meal with faba bean in practical diets on growth performance, meat quality, intestinal microbiota, and muscle transcriptomics of *Litopenaeus vannamei*. In a practical feed with fish meal, soybean meal, and flour contents of 200, 250, and 250 g/kg (control group, FB0), 150, 300, and 450 g/kg of faba bean were used to substitute 30%, 60%, and 90% of the dietary soybean meal–flour mixture (1:1; FB15, FB30, and FB45). Thus, the contents of soybean meal and flour were reduced to 175, 100, and 25 g/kg, respectively, to form four isonitrogenous feeds. Shrimp with an initial body mass of 1.40 ± 0.07 g were fed with the above four feeds for 8 weeks. All four groups presented no significant difference in growth performance, including weight gain (WG), feed conversion ratio, feed intake, and protein efficiency ratio. When faba bean inclusion reached 300 g/kg (FB30 and FB45 groups), the total free amino acid and free flavor amino acid contents in flesh were significantly increased (*p* < 0.05), and the boiling loss in the FB30 group, the steaming loss, and the boiling loss in the FB45 group were significantly lower than those of the control group (*p* < 0.05). The flesh hardness and chewing of the FB45 group were also significantly higher than those of the control group (*p* < 0.05). When faba bean inclusion reached 450 g/kg, the abundance of intestinal Proteobacteria and Actinobacteriota was decreased, while the abundance of Firmicutes was increased. In addition, the high inclusion of faba bean promoted the expression of related pathways such as myosin and myogenic fibers, as well as the genes such as fibrillin‐2 (*FBN2*), troponin C (*TnC*), and myosin regulatory light chain 2 (*MRLC2*). In conclusion, high dietary inclusion of faba bean improved the meat quality and almost completely replaced soybean meal without negative effects on the growth of Pacific white shrimp.

## 1. Introduction

Faba bean (*Vicia faba* L.) is a widely cultivated edible bean in the world, and its production reached 6 million tons in 2023, in which China accounted for about 28% of the total [1]. Faba bean contains relatively high contents of protein, carbohydrates, lysine, and vitamin C [2], and it is an important protein source for livestock and poultry feeds, such as pigs [3], cattle [4], and chickens (*Pisum sativum*) [5]. However, there are few reports on the application of faba bean in aquafeeds. In juvenile European sturgeon (*Huso huso*), dietary addition of 100 g/kg faba bean did not adversely affect growth performance, survival, hematological, and serum biochemical indices, but high inclusion (150–250 g/kg) significantly decreased the growth performance [6]. In tilapia (*Oreochromis niloticus*), the substitution of rapeseed and soybean meals with 150, 300, and 600 g/kg faba bean significantly increased the myofibers number and improved muscle hardness and chewiness [7]. Higher dietary faba bean (630–900 g/kg) significantly increased muscle myofiber area, collagen content, and crude protein content of grass carp (*Ctenopharyngodon idella*) [8]. Those findings indicated that feeding faba beans changed the meat quality of some farmed fishes. The significant improvement of flesh quality in grass carp by dietary faba bean might be concerned with the fatty acid degradation pathway and calcium signaling pathway, which exhibited significantly reduced activity, directly driving myofibrillar proliferation (increased myofibrillar density and reduced diameter) [9].

Pacific white shrimp (*Litopenaeus vannamei*) is the most significantly cultivated shrimp species worldwide, and China is the major producer. Improving the meat quality of *L. vannamei* is an important direction for shrimp farming. Considering the improved flesh quality by dietary inclusion of faba bean in some fishes, can dietary faba bean improve the flesh quality of shrimp? Therefore, in the present study, faba bean was included in feed to investigate the effects on the growth performance, muscle quality, and gut microbiota of *L. vannamei*, and the possible mechanisms were also analyzed through muscle transcriptomics. The findings will guide the application of faba bean in *L. vannamei* feed for the improvement of flesh quality.

## 2. Materials and Methods

### 2.1. Ethical Statement

The study followed the animal care protocols set by the Institutional Animal Care and Use Committee and Shanghai Ocean University’s Experimental Animal Ethics Committee.

### 2.2. Experimental Design and Diets

According to the nutritional requirements of Pacific white shrimp, a basal feed (FB0) was prepared with 200 g/kg fish meal, 250 g/kg soybean meal, and 250 g/kg wheat flour inclusion, and then, 150, 300, and 450 g/kg of faba bean were used to replace 30%, 60%, and 90% of the soybean meal–wheat flour mixture (1:1; FB15, FB30, and FB45). Therefore, the wheat flour and soybean meal content was reduced to 175, 100, and 25 g/kg, respectively, to form four isonitrogenous and isolipidic feeds. The faba bean, soybean meal, and wheat flour used in the present study contained 292.0, 442.0, and 144.1 g/kg crude protein and 35.0, 19.0, and 18.0 g/kg crude lipid, respectively. Faba bean was obtained from Nong Heyuan Agricultural Development Co. (Taizhou, China). Dried faba bean was crushed, then, sieved through 80‐mesh. After the crushing and sieving through 80‐mesh, all solid ingredients were evenly mixed with oil and water. A single screw extruder (LX‐75, Longxiang Food Machinery Factory, Hebei Province, China) was used to produce sinking pellets (diameter of 1.2 mm). Then, pellets were post‐cooked for 20 min at 95°C, allowed to air dry until the moisture content was less than 10%, then, sealed and kept for later use. The diet formula and proximate composition are presented in Table 1, and the amino acid compositions of faba bean, soybean meal, wheat flour, and experimental feeds are shown in Table 2.

**Table 1:** Formulation and proximate composition of experimental diets (air‐dried basis, g/kg).

Ingredients	FB0	FB15	FB30	FB45
Fish meal	200.0	200.0	200.0	200.0
Faba bean	0.0	150.0	300.0	450.0
Wheat flour	250.0	175.0	100.0	25.0
Soybean meal	250.0	175.0	100.0	25.0
Soy protein concentrate	100.0	100.0	100.0	100.0
Corn gluten meal	40.0	40.0	40.0	40.0
Peanut meal	50.0	50.0	50.0	50.0
Squid visceral paste	50.0	50.0	50.0	50.0
Soybean lecithin	15.0	15.0	15.0	15.0
Fish oil	15.0	15.0	15.0	15.0
Vitamin premix	5.0	5.0	5.0	5.0
Mineral premix	25.0	25.0	25.0	25.0
Total	1000.0	1000.0	1000.0	1000.0
Proximate composition				
Moisture	71.3	70.5	71.6	70.3
Crude protein	388.5	387.3	386.5	388.7
Crude lipid	67.2	68.3	68.6	69.2
Crude ash	70.7	70.4	71.2	70.3

*Note:* Each kilogram of vitamin and mineral premix contains: vitamin A, 400,000 IU; vitamin D3, 18 × 10^4^ IU; vitamin E, 3 g; vitamin K3, 1 g; vitamin B1, 0.5 g; vitamin B2, 1.5 g; vitamin B6, 0.8 g; vitamin C, 16 g; D‐biotin, 8 mg; folic acid, 0.24 g; niacinamide, 4 g; D‐calcium pantothenate, 2.5 g; inositol, 15 g; K, 42.9 g; Mg, 6 g; Fe, 36.25 g; Cu, 3.25 g; Zn, 8.5 g; Mn, 8.25 g; Co, 157 mg; Se, 25.9 mg; I, 65 mg.

**Table 2:** Amino acid composition of faba bean, soybean meal, wheat flour, and experimental feeds (dry matter basis, g/kg).

Items	FB	SBM	WF	FB0	FB15	FB30	FB45
Essential amino acids (EAAs)							
Thr	10.2	16.9	4.1	15.6	15.4	16.1	16.2
Met	2.8	6.2	1.8	5.8	5.7	5.5	5.3
Val	13.6	20.6	6.1	20.2	20.9	21.4	21.6
Ile	10.3	20.0	4,5	15.9	16.0	16.8	17.2
Leu	19.4	33.0	7.8	29.6	29.8	30.2	30.4
Phe	13.1	20.9	5.7	21.4	21.7	22.3	22.2
His	5.5	11.5	2.9	10.6	10.2	10.8	10.7
Lys	23.8	26.7	4.8	22.5	23.2	24.9	23.3
Arg	26.0	31.8	9.2	25.2	25.0	26.0	25.9
Nonessential amino acids (NEAAs)							
Asp	27.5	51.4	8.7	39.2	38.8	39.4	39.1
Ser	10.2	21.3	5.4	18.3	18.1	18.9	18.0
Glu	44.5	78.8	28.1	71.8	71.2	72.1	71.6
Gly	27.2	19.2	6.2	20.1	20.3	20.9	20.6
Ala	15.9	19.0	5.6	21.0	21.2	21.9	21.4
Cys	2.1	6.4	2.8	3.6	3.5	3.7	3.7
Tyr	7.9	15.9	3.9	10.6	10.4	11.6	11.2
Pro	9.6	21.5	17.3	14.3	14.5	14.7	14.4
Total amino acids (TAAs)	269.6	421.1	124.9	365.7	365.9	377.2	372.8

Abbreviations: FB, faba bean; SBM, soybean meal; WF, wheat flour.

### 2.3. Experimental Shrimp and Feeding Management

The feeding experiment was performed in indoor concrete pools at the Binhai Aquaculture Base of Shanghai Ocean University. Shrimp larvae were obtained from a farm located in Shanghai. During the nursing period, the initial water salinity was 5‰, then gradually reduced to 1.0‰ in 1 week. After 6 weeks of stocking with commercial feed, the larvae’s body weight was approximately 1.4 g. A total of 640 shrimps with an initial body weight of 1.40 ± 0.07 g were chosen and placed in 16 cages (1.0 m × 1.2 m × 1.0 m) with 40 shrimps per cage. All cages were placed in two concrete pools (5.0 m × 3.0 m × 1.2 m) without direct sunshine. Thus, the experiment contained four treatments with four replications per treatment.

Shrimp were fed daily at 7:00, 11:00, 17:00, and 23:00. The feeding quantity ranged from 8% (at the start) to 3% (at the end) of the total shrimp weight, and about 60% of the feed was offered in the morning and evening. The feed input was modified depending on the feeding state as well as the weather and temperature to make sure the shrimp ate up the feed within 2 h. The water temperature was 26–32°C, salinity was 0.5‰–1.0‰, dissolved oxygen was ≥5.2 mg/L, pH value was 7.6–8.7, nitrite was ≤0.05 mg/L, and ammonia nitrogen was ≤0.2 mg/L during the feeding period. The feeding trial lasted for 56 days.

### 2.4. Sampling

Before the feeding trial, 20 shrimps were combined into one sample from a total of 100 shrimps for the initial body composition analysis (stored at −20°C). Before sampling, shrimps were stopped from feeding for 24 h. Weight gain (WG) and survival were calculated based on the total weight and quantity of shrimp. Seventeen shrimp with similar sizes were selected from each cage for analytical determination. Two shrimps were stored at −20°C for body composition analysis. Four shrimps were measured for body weight, body length, hepatopancreas weight, and meat weight after shelling for calculating morphology indexes. The meat was stored at −80°C for the determination of proximate composition, amino acid composition, and biochemical indices. After blooding hemolymph from the pericardial cavity and centrifuging (4000 rpm, 10 min), the supernatant was kept at −80°C to determine the biochemical indices. For sectioning, the muscle at the sixth abdominal segment of two shrimps was sampled and immersed in GD fixative. Two shrimps were cooked for 3 min, cooled, and then the color of the second abdominal segments was measured on both sides. The water‐holding capacity was assessed by steaming, boiling fresh flesh, and thawing iced flesh. Two shrimps were taken for texture determination. Three shrimps were collected from each cage, and the surface was disinfected by 75% alcohol, then, rinsed 2–3 times with sterile saline. The muscles (2nd abdominal segment) and guts were dissected on ice trays under aseptic conditions. Following immediate freezing in liquid nitrogen, the tissues were kept at −80°C for the analysis of muscle transcriptomes and gut microbial communities.

### 2.5. Measurement Indicators and Methods

#### 2.5.1. Growth Performance and Morphological Indicators

Growth performances were calculated as follows:

Survival %=100×Initial number of shrimp/final number of shrimp.

Weight gain WG, %=100×Final average mass−initial average mass/initial average mass.

Feed intake FI, g/shrimp=Total feed intake/initial number +final number/2.

Feed conversion ratio FCR=Feeding amount/final average mass −initial average mass.

Protein efficiency rate PER, %=100×Final shrimp weight ×final shrimp crude protein −initial shrimp weight ×initial shrimp crude protein/crude protein intake.

Morphology indexes were calculated as follows:

Condition factor CF, g/cm3=100×Whole body weight/body length3.

Hepatopancreas somatic indices HSI, % =100 ×Hepatopancreas weight/whole shrimp weight.

Meat yield %=100×Muscle weight/shrimp weight.

The above measurements referred to Huang et al. [10] and Zheng et al. [11].

#### 2.5.2. Proximate Composition, Amino Acid Composition of Diets, Meat, and Shrimp

The proximate composition determination of feed, whole shrimp, and muscle was carried out using AOAC methods [12]. Moisture content was assessed by drying samples in an oven at 105°C until they reached a stable weight, while muscle moisture was determined through freeze‐drying to a constant weight. The samples were incinerated for 6 h at 550°C in a muffle furnace to determine the crude ash content. Crude lipid was measured using the chloroform–methanol method, and crude protein was measured using an automated Kjeldahl nitrogen tester (Kjeltec 2300, Foss, Sweden).

The automatic amino acid analyzer (LA8080, HITACHI, Japan) was used to determine the amount of amino acids and free amino acids in muscle or diets, following the procedure outlined by Yao et al. [13]. In brief, amino acids were determined following the hydrolysis of a 20 mg freeze‐dried sample with 6 M HCl in vacuum at 110°C for 24 h. Free amino acids were measured by extracting them from a 0.3 g fresh muscle sample using 5% trichloroacetic acid (TCA), followed by pH adjustment and filtration of the supernatant.

#### 2.5.3. Muscle Texture and Body Color

Three shrimp were selected from each cage, then carefully shelled. The muscle from the second abdominal segment was sampled for texture analysis using a TA Texture Analyzer (Shanghai Tengba Instrument Technology Co., Ltd.). Deformation was chosen as the goal mode for the 25 mm × 25 mm cylindrical probe with a test speed of 1 mm/s, a time of 2 s, and the setting variable was 35%. The indexes of determination included hardness, springiness, cohesiveness, and chewiness. Shear force was measured using the BS cutter along the vertical direction of the muscle sample from the third abdominal region.

From each cage, two shrimp were chosen to boil for 3 min and then cooled to room temperature. The WSC‐S colorimeter (Shanghai Precision Scientific Instruments Co., Ltd.) was used to measure the surface color characteristics of the second abdominal segment on both sides, including lightness (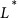), red‐greenness (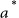) and yellow‐blueness (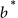).

#### 2.5.4. Water‐Holding Capacity

The approach outlined by Sun et al. [14] was used to ascertain the water‐holding capacity. In short, a block of meat (*W*_1_) was boiled or steam‐cooked for 3 min, allowed to cool, and then weighed (*W*_2_) once the surface water had been removed. After being preserved at −20°C for 24 h, another block of flesh (*W*_1_) was thawed at room temperature and weighed (*W*_2_). The indicators were calculated as follows:

Steaming boiling, thawing loss %=W1−W2/W1×100.

#### 2.5.5. Serum and Muscle Biochemical Indices

Serum biochemical indices included total protein (TP), total antioxidant capacity (T‐AOC), malondialdehyde (MDA), superoxide dismutase (SOD), total cholesterol (T‐CHO), and glutathione peroxidase (GSH‐Px). Muscle biochemical indices included GSH‐Px, T‐AOC, SOD, MDA, lactic acid (LA), and cathepsin B (Cath‐B). All the assays were performed as directed by the relevant kits’ instructions (Nanjing Jiancheng Bioengineering Institute, China).

#### 2.5.6. Muscle Histology

Following dehydration, hyalinization, and paraffin embedding, muscle samples were sectioned for hematoxylin–eosin (H&E) staining and fixed in neutral resin. An image microscope (Nikon 80i, Japan) was used to view the muscles’ morphological structure. The muscle fiber density was ascertained by counting the quantity of muscle fibers.

#### 2.5.7. Gut Microbial Community Composition

Two shrimps per cage (pooled as one sample) from the FB0 and FB45 groups were chosen for the determination of gut microbial community composition. Intestine samples were sent to Shanghai Meiji Biomedical Technology Co., Ltd. for DNA extraction and amplification of the V3–V4 variable region of the 16S rRNA gene using the Illumina MiSeq sequencing platform. The amplification primers were 338F (5′‐ACTCCTACGGGGAGGCAGCAG‐3′) and 806R (5′‐GGACTACHVGGGTWTCTAAT‐3′). Purified amplicons were polymerized on the Illumina MiSeq platform (Illumina) and paired‐end sequenced (2 × 300) according to the standard procedure of Meiji Biomedical Technology Co., Ltd. The sequencing was performed using the usparse (http://drive5.com/uparse/) for operational taxonomic unit (OTU) cluster analysis. To generate representative sequences for OTUs, chimeras were eliminated during the clustering procedure, and OTU clustering was carried out on non‐repetitive sequences. On the web platform (www.majorbio.com), the OTUs were examined to analyze community abundance and composition.

#### 2.5.8. Transcriptome Sequencing and Analysis

For transcriptome analysis, the muscle of two shrimps per cage from the FB0 and FB45 groups was combined into a sample. Total RNA was extracted using the Trizol technique (Invitrogen), and TaKara’s DNase I was used to eliminate the genomic DNA. NanoDrop2000 was used to measure the concentration and purity of RNA, and the RNA integrity number (RIN) was tested using a 2100 Bioanalyser (Agilent). Library creation and sequencing were performed by Shanghai Meiji Biomedical Technology Co. using qualified RNA.

The quantitative index was TPM (transcripts per million readings), and the gene expression levels were measured using the expression quantification software RSEM. The DESeq2 method was used to look for differentially expressed genes (DEGs) between samples. The multiplicity of difference was defined as |log_2_FC| ≥ 1, and the difference value was defined as *p*‐value <0.05. All DEGs underwent KEGG annotation and pathway enrichment analysis, GO annotation, and functional enrichment analysis. Fisher’s method was used to test the precision of each analysis, and the BH method was used to adjust for the *p*‐value.

#### 2.5.9. Statistical Analysis

Data were expressed as mean ± standard deviation. SPSS 26.0 statistical analysis software (SPSS Inc., Michigan Avenue, Chicago, IL, USA) was used for data processing and analysis. All data were subjected to the one‐way analysis of variance (ANOVA) test. Multiple group comparisons were conducted using Tukey’s multirange test. A difference of *p* < 0.05 was deemed significant.

## 3. Results

### 3.1. Growth Performance

As shown in Table 3, there was no significant difference (*p* > 0.05) in survival, FW, FI, WG, FCR, CF, HSI, meat yield, and PER among all the groups.

**Table 3:** Dietary effects of faba bean on growth performance and body indices of *Litopenaeus vannamei*.

Groups	FB0	FB15	FB30	FB45
IW (g)	1.40 ± 0.07	1.40 ± 0.09	1.40 ± 0.07	1.40 ± 0.12
FW (g)	10.41 ± 0.93	10.73 ± 0.07	10.25 ± 0.44	10.11 ± 0.49
Survival (%)	86.3 ± 3.2	88.8 ± 3.2	92.5 ± 3.5	91.9 ± 3.1
FI (g/shrimp)	14.39 ± 0.25	14.20 ± 0.24	13.93 ± 0.25	14.08 ± 0.23
WG (%)	638.3 ± 46.1	660.8 ± 5.1	627.1 ± 31.2	636.5 ± 11.5
FCR	1.45 ± 0.08	1.46 ± 0.01	1.49 ± 0.08	1.50 ± 0.06
HSI (%)	3.32 ± 0.31	3.32 ± 0.33	3.38 ± 0.36	3.44 ± 0.32
CF (g/cm^3^)	0.62 ± 0.05	0.65 ± 0.05	0.63 ± 0.04	0.65 ± 0.05
Meat yield (%)	52.48 ± 1.91	51.47 ± 2.33	51.87 ± 1.75	53.30 ± 1.77
PER (%)	35.21 ± 1.63	36.22 ± 1.86	35.83 ± 1.65	35.92 ± 0.26

Abbreviations: CF, condition factor; FCR, feed conversion ratio; FI, feed intake; FW, final weight; HSI, hepatopancreas‐somatic index; IW, initial weight; PER, protein efficiency rate; WGR, weight gain rate.

### 3.2. Muscle Texture, Water‐Holding Capacity, and Body Color

In Table 4, there were no significant differences in cohesiveness, springiness, shear force, thawing loss, and body color among the four groups (*p* > 0.05). The FB45 group showed significantly higher chewiness and hardness, as well as lower boiling and steaming loss (*p* < 0.05) compared to the control group. The boiling loss of the FB30 group was also significantly lower than that of the control (*p* < 0.05).

**Table 4:** Dietary effects of faba bean on muscle texture, water‐holding capacity, and body color of *Litopenaeus vannamei*.

Groups	FB0	FB15	FB30	FB45
Muscle texture				
Hardness (gf)	742.64 ± 65.05^b^	616.78 ± 45.72^b^	648.12 ± 51.27^b^	933.52 ± 32.53^a^
Springiness (gf)	0.60 ± 0.05	0.58 ± 0.02	0.60 ± 0.05	0.63 ± 0.04
Cohesiveness (gf)	0.54 ± 0.04	0.52 ± 0.04	0.58 ± 0.02	0.58 ± 0.04
Chewiness (gf)	238.63 ± 23.07^b^	227.42 ± 21.55^b^	237.95 ± 18.85^b^	392.18 ± 38.60^a^
Shear force (gf)	761.14 ± 66.60	791.81 ± 78.50	814.55 ± 80.08	806.48 ± 77.10
Water‐holding capacity				
Steaming loss (%)	21.21 ± 0.95^a^	20.04 ± 0.89^a,b^	20.00 ± 1.30^a,b^	19.24 ± 0.44^b^
Boiling loss (%)	26.63 ± 2.18^a^	24.55 ± 1.02^a,b^	23.02 ± 1.88^b^	22.23 ± 1.66^b^
Thawing loss (%)	3.18 ± 0.25	3.14 ± 0.08	3.11 ± 0.15	3.20 ± 0.05
Body color				
Lightness ( 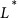 )	50.51 ± 2.55	49.80 ± 2.76	50.50 ± 1.19	49.06 ± 2.11
Red‐greenness ( 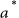 )	21.40 ± 1.92	23.08 ± 1.66	24.29 ± 2.17	22.83 ± 1.38
Yellow‐blueness ( 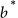 )	31.64 ± 2.37	33.76 ± 2.86	33.24 ± 3.04	31.26 ± 2.43

*Note:* Data with different letters in the same row indicate significant differences (*p* < 0.05).

### 3.3. Whole Shrimp and Muscle Composition

As shown in Table 5, the FB45 group presented significantly higher crude lipid content in muscle than the control group (FB0), the FB15 group, and the FB30 group (*p* < 0.05). There was no significant difference in whole body composition and muscle moisture, crude protein, and crude ash contents among all the groups (*p* > 0.05).

**Table 5:** Dietary effects of faba bean on whole shrimp and muscle composition of *Litopenaeus vannamei* (fresh weight, g/kg).

Groups	FB0	FB15	FB30	FB45
Whole body				
Moisture	741.32 ± 3.84	748.27 ± 12.60	758.44 ± 10.38	743.20 ± 4.50
Crude protein	203.09 ± 2.44	199.50 ± 6.73	195.03 ± 8.95	205.28 ± 7.48
Crude lipid	16.17 ± 1.33	16.20 ± 1.52	16.21 ± 0.59	16.15 ± 0.38
Crude ash	26.44 ± 0.97	28.90 ± 1.68	25.66 ± 1.86	27.50 ± 1.90
Muscle				
Moisture	767.26 ± 13.76	772.76 ± 18.35	776.41 ± 17.43	757.77 ± 26.21
Crude protein	188.71 ± 10.06	192.81 ± 13.31	190.09 ± 12.61	206.58 ± 3.78
Crude lipid	11.20 ± 0.20^b^	10.94 ± 0.25^b^	11.24 ± 0.25^b^	12.15 ± 0.59^a^
Crude ash	12.70 ± 0.70	13.35 ± 0.66	13.76 ± 0.73	14.12 ± 0.53

*Note:* Data with different letters in the same row indicate significant differences (*p* < 0.05).

### 3.4. Muscle Amino Acid Content

As shown in Table 6, there was no significant difference (*p* > 0.05) in muscle amino acid contents among all the groups.

**Table 6:** Dietary effects of faba bean on amino acids composition in muscle of *Litopenaeus vannamei* (dry matter basis, g/kg).

Items	FB0	FB15	FB30	FB45
Essential amino acids (EAAs)				
Thr	32.58 ± 2.59	31.12 ± 2.76	31.37 ± 1.35	32.44 ± 0.36
Met	16.65 ± 1.05	16.94 ± 0.82	16.09 ± 0.44	16.66 ± 0.29
Val	41.37 ± 3.22	40.19 ± 0.66	40.33 ± 0.84	41.93 ± 0.59
Ile	39.19 ± 0.86	39.51 ± 1.31	40.16 ± 0.79	40.48 ± 1.55
Leu	61.56 ± 0.15	62.19 ± 0.15	61.84 ± 0.83	61.80 ± 0.30
Phe	40.16 ± 2.71	40.58 ± 3.80	40.95 ± 0.75	41.43 ± 0.92
His	16.82 ± 0.70	17.11 ± 1.68	17.58 ± 0.65	17.83 ± 0.41
Lys	73.04 ± 1.93	72.89 ± 2.51	73.49 ± 0.86	73.97 ± 1.04
Arg	78.17 ± 4.48	77.43 ± 4.11	79.25 ± 2.74	79.41 ± 1.12
Nonessential amino acids (NEAAs)				
Asp	83.24 ± 3.06	83.21 ± 7.79	85.69 ± 3.53	85.39 ± 3.74
Ser	31.86 ± 2.16	31.38 ± 1.57	31.85 ± 1.25	31.82 ± 0.38
Glu	144.09 ± 3.87	143.82 ± 6.92	145.25 ± 6.50	145.9 ± 10.46
Gly	72.28 ± 5.64	71.67 ± 2.72	73.25 ± 1.18	72.8 ± 2.37
Ala	49.82 ± 4.56	50.07 ± 4.55	51.8 ± 1.30	52.01 ± 1.58
Cys	7.96 ± 0.66	7.81 ± 0.55	8.02 ± 0.34	8.52 ± 0.31
Tyr	25.77 ± 1.30	25.72 ± 1.38	27.23 ± 1.64	27.73 ± 1.39
Pro	46.52 ± 1.16	45.27 ± 2.14	46.34 ± 2.11	47.75 ± 2.83
TAAs	861.09 ± 31.31	856.9 ± 32.86	870.5 ± 9.48	877.84 ± 11.31

Abbreviation: TAAs, total amino acids.

In Table 7, a total of 17 free amino acids were detected in muscle, with the highest content of Gly, followed by Pro and Arg. The total free amino acid level of the FB30 and the FB45 groups and the flavor amino acid level of the FB30 group were significantly higher than those of the control group (*p* < 0.05).

**Table 7:** Dietary effects of faba bean on free amino acid composition in muscle of *Litopenaeus vannamei* (fresh weight, mg/100 g).

Items	FB0	FB15	FB30	FB45
Asp ^∗^	14.43 ± 0.18^b^	14.84 ± 0.58^b^	16.12 ± 0.31^a^	15.53 ± 0.46^a,b^
Thr	89.28 ± 1.27	89.93 ± 1.08	90.38 ± 4.96	88.28 ± 2.99
Ser	25.38 ± 2.07	23.89 ± 0.76	23.61 ± 2.29	24.2 ± 1.60
Glu ^∗^	36.70 ± 1.12^c^	52.40 ± 5.28^a,b^	54.88 ± 2.83^a^	44.62 ± 4.92^b,c^
Gly ^∗^	845.42 ± 54.55	889.50 ± 32.41	932.67 ± 67.83	890.55 ± 35.60
Ala ^∗^	82.68 ± 6.42^c^	109.98 ± 8.86^a,b^	112.25 ± 8.61^a^	94.32 ± 0.51^b,c^
Cys	4.07 ± 0.02	4.16 ± 0.20	4.23 ± 0.14	3.91 ± 0.05
Val	20.26 ± 1.64	20.97 ± 0.84	22.19 ± 2.06	20.49 ± 0.73
Met	4.30 ± 0.30	5.71 ± 0.41	6.17 ± 0.78	4.70 ± 0.37
Ile	6.83 ± 0.62	6.97 ± 0.21	7.96 ± 0.35	7.01 ± 0.34
Leu	13.88 ± 1.02	13.95 ± 0.41	15.85 ± 0.58	13.26 ± 0.65
Tyr ^∗^	16.55 ± 1.07	15.23 ± 1.05	15.51 ± 0.99	15.43 ± 1.47
Phe ^∗^	12.18 ± 0.59	11.58 ± 0.20	12.81 ± 0.82	11.97 ± 1.09
Lys	35.38 ± 3.01	34.76 ± 1.29	36.32 ± 3.65	37.41 ± 1.58
His	22.10 ± 1.66	20.81 ± 1.92	21.04 ± 1.41	19.94 ± 1.87
Arg	593.04 ± 57.64	535.15 ± 16.37	552.65 ± 31.49	597.34 ± 8.53
Pro	825.43 ± 21.04^b^	827.83 ± 30.51^b^	902.59 ± 30.65^a,b^	931.49 ± 31.15^a^
DAAs	1007.96 ± 48.82^b^	1093.53 ± 44.49^a,b^	1144.23 ± 64.88^a^	1072.41 ± 32.40^a,b^
TAAs	2647.89 ± 81.12^b^	2677.66 ± 12.55^b^	2827.21 ± 34.35^a^	2820.44 ± 47.00^a^

*Note:* DAAs: flavor amino acids, include Asp, Glu, Gly, Ala, Tyr, Phe, labeled with  ^∗^. Data with different letters in the same row indicate significant differences (*p* < 0.05).

Abbreviation: TAAs, total amino acids.

### 3.5. Hemolymph and Muscle Biochemical Indices

In hemolymph, there were no significant differences in TP, MDA content, and GSH‐Px activity among all the groups (*p* > 0.05). The FB15 group showed significantly higher T‐CHO content than the control (*p* < 0.05). The SOD activity and T‐AOC were significantly higher in the FB30 group than in those in the control (*p* < 0.05; Table 8).

**Table 8:** Dietary effects of faba bean on hemolymph and muscle biochemical indices of *Litopenaeus vannamei*.

Items	FB0	FB15	FB30	FB45
Hemolymph				
TP (gprot/L)	28.21 ± 0.46	28.58 ± 1.29	31.71 ± 2.50	29.44 ± 2.71
MDA (nmol/ml)	4.30 ± 0.36	2.96 ± 0.22	3.66 ± 0.27	4.49 ± 0.38
SOD (U/ml)	204.55 ± 20.03^c^	245.43 ± 19.34^a,b,c^	301.41 ± 4.51^a^	271.36 ± 13.86^a,b^
T‐AOC (U/gprot)	115.13 ± 9.47^b^	129.50 ± 10.77^a,b^	136.53 ± 8.13^a^	119.02 ± 9.79^a,b^
T‐CHO (mmol/L)	2.88 ± 0.10^b^	3.82 ± 0.13^a^	3.25 ± 0.23^b^	3.53 ± 0.31^a,b^
GSH‐Px (U/mgprot)	318.00 ± 24.04	330.76 ± 19.10	365.48 ± 34.46	356.95 ± 18.95
Muscle				
MDA (nmol/mgprot)	2.27 ± 0.18^a^	2.10 ± 0.05^a,b^	1.99 ± 0.11^a,b^	1.68 ± 0.13^b^
SOD (U/mgprot)	220.09 ± 13.97^b^	248.87 ± 20.82^a,b^	279.92 ± 14.10^a^	261.66 ± 9.27^a^
T‐AOC (U/gprot)	8.66 ± 0.76	10.43 ± 0.56	10.19 ± 0.91	9.57 ± 0.92
GSH‐Px (U/mgprot)	150.68 ± 4.78	176.59 ± 1.64	158.72 ± 9.02	150.54 ± 7.31
LA (nmol/mgprot)	51.37 ± 4.86	57.66 ± 4.29	51.09 ± 0.41	54.31 ± 2.87
Cath‐B (ng/g)	70.59 ± 6.23	71.18 ± 3.71	73.42 ± 4.27	77.53 ± 7.72

*Note:* Data with different letters in the same row indicate significant differences (*p* < 0.05).

Abbreviations: Cath‐B, cathepsin B; GPx, glutathione peroxidase; LA, lactic acid; MDA, malondialdehyde; SOD, superoxide dismutase; T‐AOC, total antioxidant capacity; T‐CHO, total cholesterol; TP, total protein.

In muscle, no significant difference was observed in T‐AOC, LA content, GSH‐Px, and Cath‐B activity among all the groups (*p* > 0.05). The MDA content was decreased, and SOD activity was increased with the increasing faba bean inclusion. The MDA content in the FB45 group was significantly lower, and the SOD activity in the FB30 and FB45 groups was significantly higher than those in the control (*p* < 0.05; Table 8).

### 3.6. Muscle Histology

Muscle fiber structure and fiber density are shown in Figure 1 and Table 9, respectively. The myofiber density of the FB45 group was significantly higher than that of the other groups (FB0, FB15, and FB30 groups; *p* < 0.05).

Figure 1Effect of dietary faba bean on muscle fiber structure of *Litopenaeus vannamei* (H&E staining). (A–D) FB0, FB15, FB30, and FB45 groups.

**Figure:**
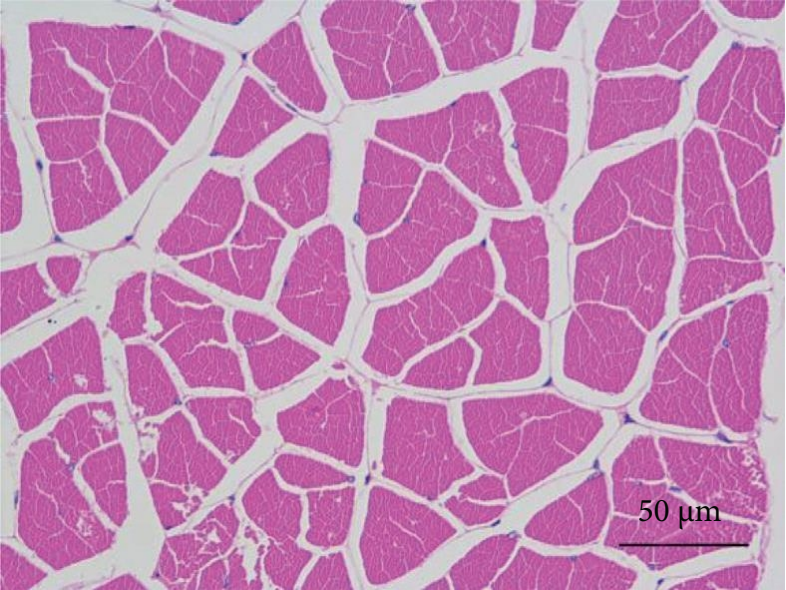
(A)

**Figure:**
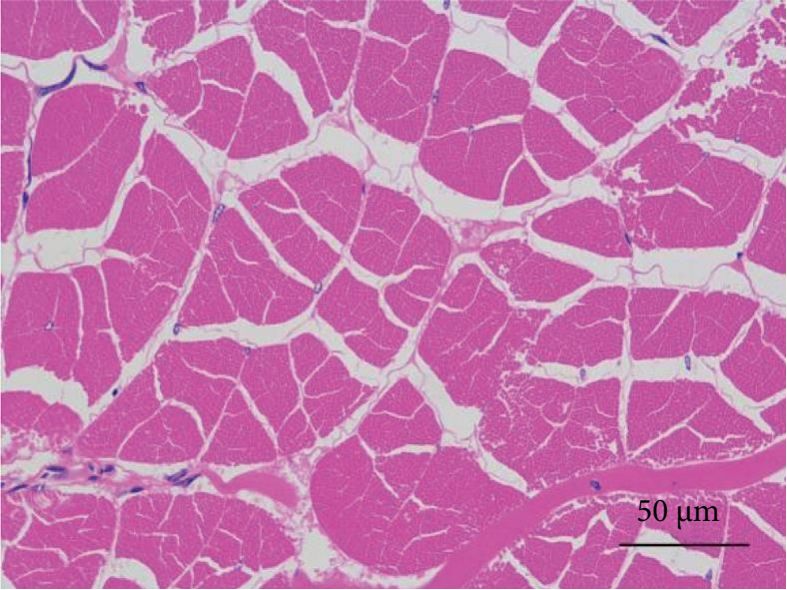
(B)

**Figure:**
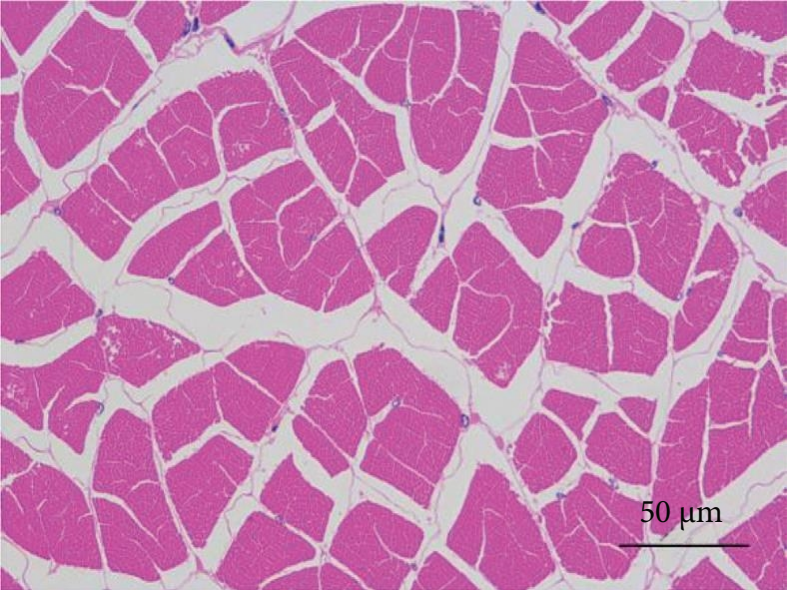
(C)

**Figure:**
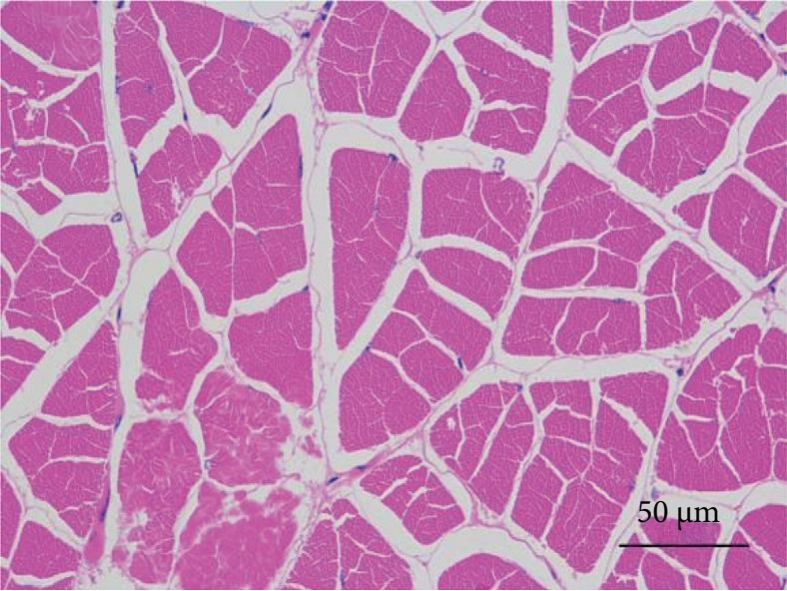
(D)

**Table 9:** Dietary effects of faba bean on myofiber density of *Litopenaeus vannamei*.

Items	FB0	FB15	FB30	FB45
Myofiber density (cell/mm^2^)	233.44 ± 0.91^b^	232.95 ± 0.56^b^	233.11 ± 2.70^b^	267.54 ± 12.36^a^

*Note:* Data with different letters in the same row indicate significant differences (*p* < 0.05).

### 3.7. Gut Microbial Community Composition

**Figure 2:**
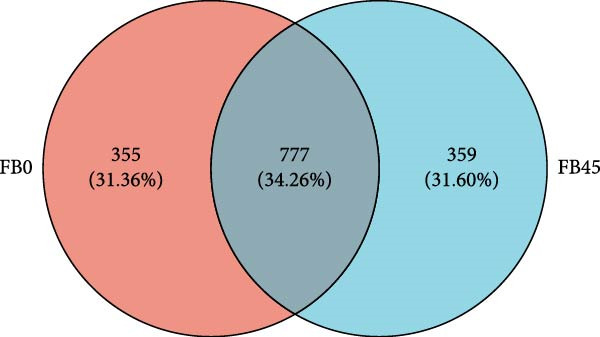
Venn diagram of gut microbial OTUs between the FB45 and the FB0 groups.

A total of 745,950 sequences were obtained from the intestinal microorganisms. The coverage index of each sample sequence read was greater than 0.999, indicating that the microbial community was adequately sampled and the data were representative. As shown in the Venn diagram of OTUs (Figure 2), a total of 1491 OTUs were found, and the FB0 and FB45 groups shared the common OTUs of 777 with 355 and 359 specific OTUs for the FB0 and FB45 groups.

**Figure 3:**
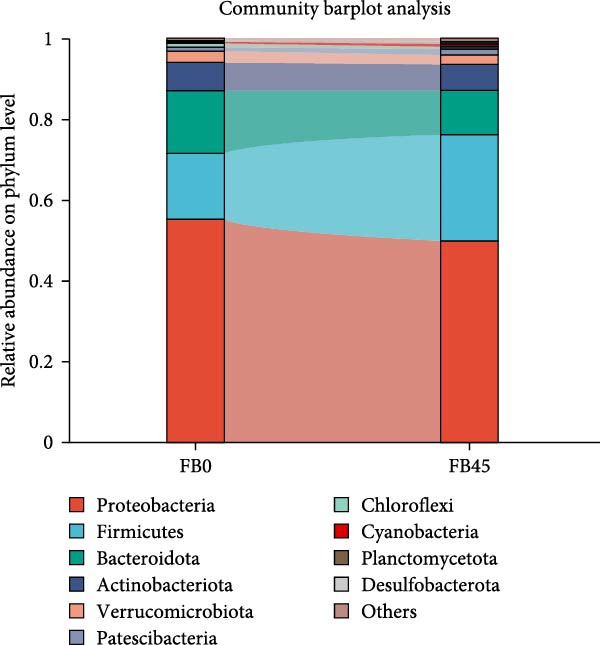
Relative abundance of gut bacterial community at phylum level.

At the phylum level (Figure 3), Proteobacteria and Bacteroidota were the major communities of intestinal microorganisms. In the FB0 group, the abundance of Proteobacteria accounted for 55.25%, followed by Firmicutes (16.35%), Bacteroidota (15.36%), and Actinobacteriota (7.10%). Compared to the FB0 group, the abundance of Proteobacteria, Bacteroidota, and Actinobacteriota in the FB45 group was decreased, while the abundance of Firmicutes was increased.

**Figure 4:**
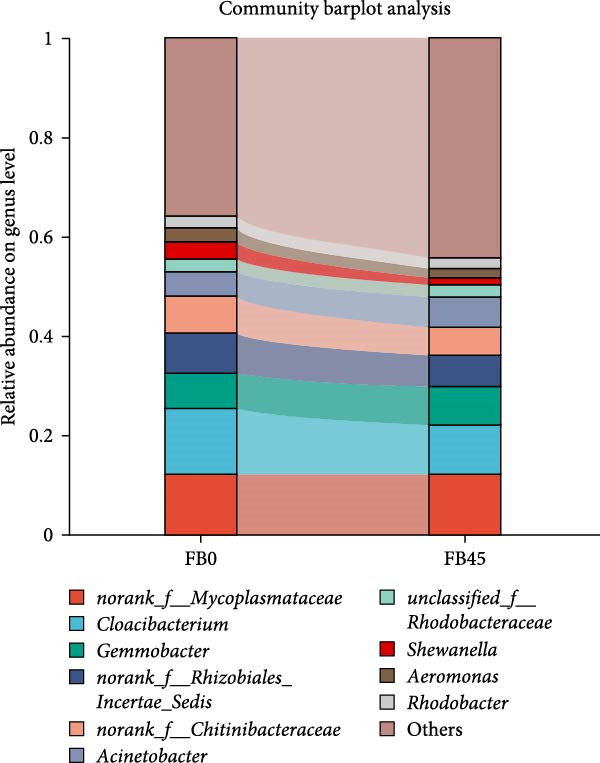
Relative abundance of gut bacterial community at genus level.

At the genus level (Figure 4), gut microorganisms mainly consisted of *Cloacibacterium*, *norank_f__Mycoplasmataceae*, and *norank_f__Rhizobiales_Incertae_Sedis*. The dominant organisms in the FB0 group were *Cloacibacterium* (13.29%), *norank_f__Mycoplasmataceae* (12.16%), *norank_f__Rhizobiales_Incertae_Sedis* (8.07%), and *Gemmobacter* (7.08%). In the FB45 group, *norank_f__Mycoplasmataceae* (12.24%) was the most dominant organism with the highest percentage, while *Cloacibacterium* decreased to 9.94% and *Gemmobacter* increased to 7.69%.

### 3.8. Muscle Transcriptome

#### 3.8.1. Quantitative Analysis of DEGs

**Figure 5:**
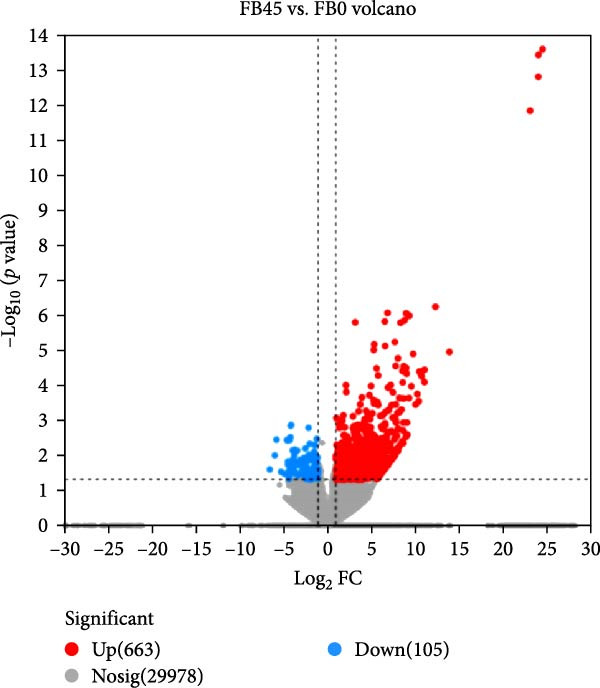
Volcano plot of differentially expressed genes between the FB45 and the FB0 groups. The vertical line is the fold change between two samples, and the horizontal line represents the *p*‐value. Each point represents a specific gene; red dots and blue dots represent significantly upregulated and downregulated genes, and gray dots are nonsignificantly different genes.

The DEGs between the FB45 and the FB0 groups are shown in Figure 5. Compared with the FB0 group, there were 768 DEGs in the FB45 group with 663 upregulated and 105 downregulated.

#### 3.8.2. GO Function Annotation Analysis

**Figure 6:**
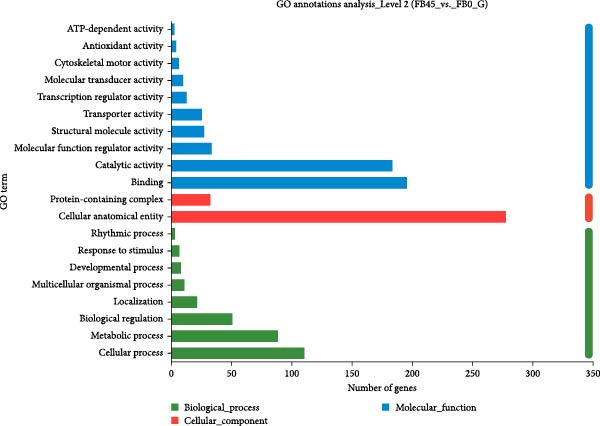
GO classification statistics column chart of DEGs between the FB45 and the FB0 groups. Histogram of GO classification statistics (multiple gene sets): in the graph, the ordinate indicates the secondary classification and terminology of GO, the abscissa indicates the number of genes/transcripts in the secondary classification, and the colors indicate different gene sets.

The DEGs obtained by comparing the FB45 group with the FB0 group were analyzed by GO annotation, and the results are shown in Figure 6. The three categories (molecular function, cellular component, and biological process) in the GO database have multiple secondary classifications, in which all DEGs were assigned to 26 secondary classifications, nevertheless. In Figure 6, only 20 secondary classifications with comparatively high quantities were displayed. In the 26 secondary classifications, 13 classifications were categorized into molecular function categories, including binding, catalytic activity, molecular function regulator activity, and antioxidant activity, and two classifications were categorized into cellular components, including protein‐containing complex and cellular anatomical entity. Another 11 classifications were categorized into the biological process category, including biological regulation, cellular process, and metabolic process.

#### 3.8.3. GO Function Enrichment Analysis

**Figure 7:**
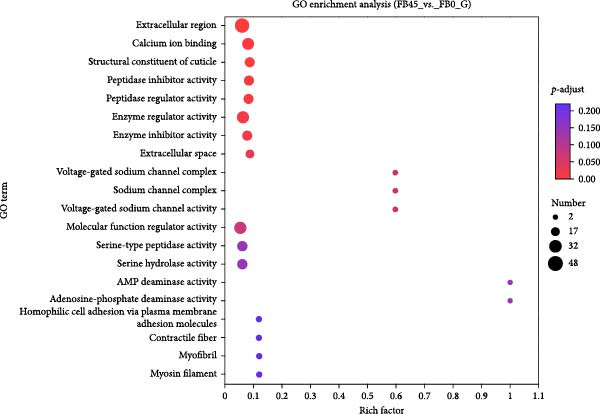
GO enrichment analysis bubble diagram of DEGs between the FB45 and the FB0 groups. The vertical axis represents GO term and the horizontal axis represents rich factor (the ratio of the number of genes/transcripts enriched in the GO term (sample number) to the number of annotated genes/transcripts (background number). The larger the rich factor, the greater the enrichment degree; the size of the dot indicates the number of genes/transcripts in this GO term, and the color of the dot corresponds to different *p*‐adjust ranges.

The GO enrichment analysis was performed based on GO functional annotation, and the results are shown in Figure 7. The DEGs were significantly enriched in the extracellular region, calcium ion binding, peptidase regulator activity, enzyme regulator activity, enzyme inhibitor activity, and extracellular space. The signaling pathways of contractile fiber, myofibril, and myosin filament were also enriched.

#### 3.8.4. KEGG Functional Annotation Analysis

**Figure 8:**
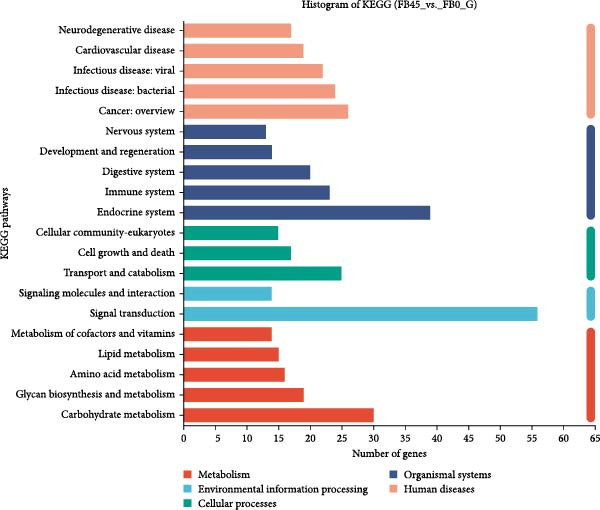
KEGG pathway classification statistical histogram of DEGs between the FB45 and the FB0 groups. The ordinate is the name of the KEGG metabolic pathway; the abscissa is the number of genes or transcripts annotated to this pathway.

The DEGs obtained by comparing the FB45 group with the FB0 group were subjected to KEGG signaling pathway annotation analysis, and the results are shown in Figure 8. All the DEGs were annotated in three categories of KEGG metabolism pathways, including environmental information processing, cellular processes, organismal systems, human diseases, and metabolism. Among them, the highest number of differential genes was signal transduction and endocrine system, followed by cancer: overview and carbohydrate metabolism, then transport and catabolism and infectious diseases: bacterial.

#### 3.8.5. KEGG Functional Enrichment Analysis

**Figure 9:**
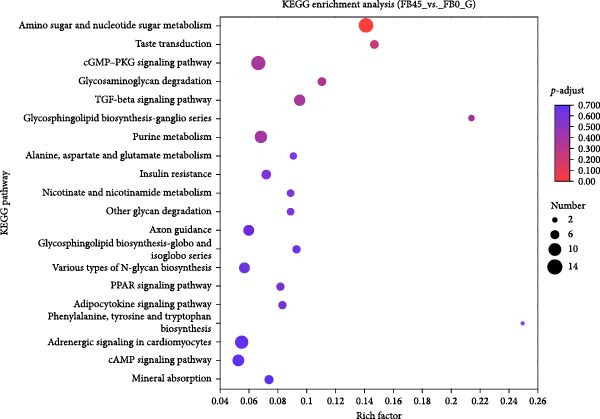
KEGG signal pathway enrichment analysis bubble diagram of DEGs between the FB45 and the FB0 groups. The vertical axis represents KEGG signaling pathways and the horizontal axis represents the rich factor. The rich factor is defined as the ratio of the number of DEGs enriched in the target KEGG pathway to the total number of annotated genes in the target KEGG pathway; a larger rich factor indicates a higher degree of enrichment of DEGs in that pathway. The size of each dot corresponds to the number of DEGs enriched in the corresponding pathway. The color of each dot corresponds to different ranges of *p*‐adjust values, with the significance threshold set at *p*‐adjust <0.05.

KEGG enrichment analysis showed that DEGs were enriched into 293 signaling pathways between the FB45 group and the FB0 group, and the bubble plot displayed the 20 signaling pathways with the highest degree of enrichment (Figure 9). In contrast to the FB0 group, DEGs in the FB45 group were significantly enriched in the amino sugar and nucleotide sugar metabolism signaling pathway. The cGMP‐PKG signaling pathway, glycosaminoglycan degradation, and the TGF‐beta signaling pathway were also enriched.

## 4. Discussion

### 4.1. Growth Performance

Due to the unbalanced amino acid composition, high starch and low fat levels, and the presence of antinutritional factors, feeding fish with faba beans would negatively affect the growth performance [15]. When grass carp were fed with soaked faba bean for 77 days, the WG and protein efficiency were significantly lower than those of fish fed a compound diet [16]. The similar result was also reported by Tian et al. [17] for grass carp fed with faba bean for 120 days. When 40%–70% of faba bean was included in the diet of tilapia for 90 days, the WG and specific growth rate were also significantly reduced [18]. The adverse effects of faba bean can be effectively reduced if the amount of faba bean used is controlled or mixed with other ingredients. Ouraji et al. [19] found that rainbow trout could tolerate 30% of faba bean in the feed, but the growth performance of the 30% faba bean group was significantly lower than that of the 15% faba bean group. Gan et al. [20] reported that replacing soybean meal with 420.3 g/kg faba bean did not affect the growth and feed utilization of grass carp, but the higher level resulted in growth retardation. However, in European seabass (*Dicentrarchus labrax*), the inclusion of 15% faba bean significantly increased the digestibility of protein, fat, starch, and energy [21]. In this study, replacing 225 g/kg soybean meal +225 g/kg wheat flour with 450 g/kg faba bean meal did not affect the WG and feed conversion ratio of shrimp. Whether higher inclusion of faba bean would adversely affect the growth performance of Pacific white shrimp requires further studies.

Faba bean seeds and skins contain phenolic substances such as flavonoids and anthocyanins, which have antioxidant and hypoglycemic effects, as well as strong bacterial inhibition against *Escherichia coli* and *Salmonella* [22]. MDA is the oxidized product of free radical peroxidation in the organism [23], which can reflect the peroxidation and cellular damage degree [24]. SOD is an essential enzyme in the biodefense system [25], which can scavenge superoxide anion reduce, the damage caused by free radicals [26], and react to the antistress properties of shrimp [27]. T‐AOC is usually employed as a crucial metric to evaluate the antioxidant capability of the organism [28]. GSH‐Px is an important peroxidative catabolic enzyme that acts as a protector of cellular membrane structure [29]. In this study, with the increase of faba bean in diets, the MDA content decreased and the SOD activity increased in shrimp muscle. The inclusion of 30% faba bean significantly increased the SOD activity and T‐AOC in hemolymph, and GSH‐Px activity also showed an upward trend. The production and transfer of free radicals can be efficiently inhibited by the antioxidant enzymes in the muscle, which can also prevent the loss of muscle tenderness, nutritional value, and organoleptic quality [30]. The improved antioxidant capacity may be related to the phenolic substances, such as flavonoids and anthocyanins, in faba beans. Yan et al. [31] found that crude extracts of proanthocyanidins from faba bean shells possessed excellent antioxidant capacity to scavenge free radicals (–DPPH and –OH) and resist lipid peroxidation as ascorbic acid and 2,6‐di‐tert‐butyl‐4‐methylphenol (BHT). Additionally, soy isoflavones also have the function of health care and preventing diseases, especially the antitumor effect [32].

### 4.2. Muscle Quality

Water‐holding capacity is an indicator reflecting the ability of muscle tissue to retain water. Moisture retention helps maintain tenderness and reduce the loss of flavor substances (e.g., amino acids and fatty acids). Crude fat content affects the friction between muscle bundles and muscle tenderness, and low fat content in meat usually means poor tenderness [33]. In this study, the boiling loss in the FB30 group and the steaming loss and boiling loss in the FB45 group were significantly lower than those of the control. The crude fat content of shrimp muscle increased significantly when faba bean inclusion reached 45%, which was different from the results on grass carp [34] and channel catfish (*Ictalurus punctatus*) [35], but similar to the results on tilapia [36]. The different results may be related to the species of aquatic animals, the feeding period, and the feeding amount of faba bean.

One important source of flavor in meat is the free flavor amino acids, such as Glu, Asp, Ala, and Gly. In this study, free total amino acid content in muscle was significantly increased by the inclusion of 30% or 45% faba bean, and the FB30 group also showed significantly higher free flavor amino acid content than the control, which indicated that the flavor of shrimp meat was improved by dietary inclusion of faba bean. Such results were similar to the findings in grass carp [37].

Textural property is the characteristic parameter measured by a texture analyzer, including hardness, elasticity, cohesion, chewiness, and so forth. [38]. Among them, hardness is closely related to other physical parameters and usually used as a basic quality indicator to reflect the texture, flavor, and economic value of meat [39]. In this study, the hardness and chewiness of shrimp muscles were significantly higher in the FB45 group than those in the control, consistent with the findings in grass carp [40], channel catfish [35], and tilapia [36]. As the basic unit of muscle structure, muscle fiber is an important factor in determining muscle texture. Generally, there is a direct relationship between muscle fiber diameter and meat hardness, and a smaller diameter of muscle fiber means higher density and harder texture [41]. Yu et al. [9] found that the diameter of muscle fiber of grass carp decreased significantly with the prolonging time of feeding faba bean, which might be related to the reduced gene expression of myosin and actin [9] and downregulation of phosphorylation of promyosin [42]. The reduced myosin and actin expression will decrease myosin ATPase activity and further impair the actin–myosin interactions [43]. The downregulation of phosphorylation of promyosin will promote the tighter binding of muscle thin filaments and thick filaments [44]. The proportion differences of muscle fiber types can lead to the differences in physicochemical properties of muscle, such as texture, color, and water‐holding capacity. The key signaling molecules of the Wnt/*β*‐catenin signaling pathway are closely associated with muscle hyperplasia, and their expression levels showed a significant upregulation trend in crisp grass carp. The changes in expression pattern may promote the myofiber quantity by regulating the molecular regulatory network of myogenesis [9]. In this study, the FB45 group presented significantly higher myofiber density than the control, which may be an important reason for the elevated textural properties. It is speculated that the improved meat quality by faba bean was regulated through myofibrillar proteins, and the relevant bioactive components in faba bean might exert effects on the muscle hyperplasia‐related pathways in shrimp.

There are three types of proteins in meat: myofibrillar proteins, myoplasmic proteins, and matrix proteins. Myofibrillar proteins are the proteins maintaining the structure of muscle, and a higher content of myofibrillar proteins represents higher elasticity of muscle [45]. The high fraction of matrix proteins is usually associated with higher muscle hardness [46]. Myoplasmic proteins are composed of proteins present in the cytoplasm of myogenic fibers and various proteases in metabolism [47]. Godiksen et al. [48] found that the mass fraction of histone proteases derived from myoplasmic proteins was correlated with the flesh texture of rainbow trout (*Oncorhynchus mykiss*). Wu et al. [37] reported that crisp grass carp showed significantly higher contents of myoplasmic proteins, myofibrillar, and matrix proteins than common grass carp. Whether feeding shrimp with faba bean affects the protein component in muscles needs further study.

### 4.3. Gut Microorganisms

Gut microorganisms play crucial roles in the immune function and in preventing the invasion of pathogens [49], and they are important indicators for evaluating the health status of aquatic animals [50]. In this study, the replacement of soybean meal‐wheat flour by faba bean increased the abundance of Firmicutes and decreased the abundance of Proteobacteria, Bacteroidota, and Actinobacteriota. In grass carp, feeding faba bean decreased the abundance of Firmicutes and Actinobacteriota and increased the abundance of Proteobacteria in the gut [51]. This difference may be related to the differences in species, water quality, and the form and proportion of faba bean. Proteobacteria are gram‐negative bacteria with an outer membrane composed of lipopolysaccharide. Lipopolysaccharide is an endotoxin that can cause fever, microcirculatory disorders, and endotoxic shock, and it is also associated with intestinal inflammation and metabolic disorders [52]. Some studies have shown that the increase of Proteobacteria number caused imbalanced intestinal flora and inflammatory response in animals [53]. Therefore, a high percentage of Proteobacteria in intestinal flora would induce diseases detrimental to intestinal health. Whereas, most of the Firmicutes phylum are gram‐positive and play key roles in host nutrition and metabolism through short‐chain fatty acid synthesis [54]. Generally, the Firmicutes phylum are considered the main microbiota in a healthy gut. Thus, the decrease of Proteobacteria and the increase of Firmicutes are favorable to the intestinal health of shrimp.

### 4.4. Muscle Transcriptome

The PPAR gene family mainly affects adipocyte differentiation by regulating the expression of genes involved in cellular lipid metabolism. When the PPAR signaling pathway is activated, it can initiate the process of adipose synthesis by regulating the expression of adipose synthesis‐related genes, which makes PPAR a key signaling factor for adipose synthesis [55]. In this study, the DEGs of PPAR signaling pathway were upregulated in the FB45 group, and the FB45 group also presented higher crude lipid content in muscle than the control, which may be related to the regulation of the PPAR signaling pathway. The enriched pathways in the FB45 group also included myosin filament, myofibril, and contractile fiber, which are all involved in the regulation of muscle growth and development or myofibril assembly. In addition, the significant upregulation of fibrillin‐2 (*FBN2*), troponin C (*TnC*), and myosin regulatory light chain 2 (*MRLC2*) genes was found in KEGG‐enriched pathway maps. FBNs are structural elements of extracellular calcium‐binding microfibrils, and microfibrils, including FBN2, control the initial stages of elastic fiber assembly. By regulating TGF‐*β* bioavailability and adjusting TGF‐*β* and BMP levels, respectively, FBN2 can control the osteoblast maturation [56]. Accompanying tropomyosin on the actin filament, troponin is a key regulatory protein of striated muscle contraction. Tn consists of three subunits: *TnI*, *TnT*, and *TnC*. *TnI* is the inhibitor of actomyosin ATPase, and *TnT* contains the binding site for tropomyosin. The inhibitory effect of *TnI* is eliminated when calcium binds to *TnC*, enabling actin–myosin interaction, ATP hydrolysis, and tension production [57]. *MRLC2* primarily regulates the myosin activity [58]. As the most prevalent protein in muscle, myosin supplies energy for muscle contraction and is involved in cytoplasmic division, cell polarity, intracellular transport, transcriptional control, and signal transduction [59]. Myosin makes up the majority of the muscular cytoskeleton, and its quantity is positively connected with muscle hardness [60]. Faba bean may alter muscle quality by affecting the expression of myosin and myofibril‐related genes. Xu et al. [44] analyzed the changes of muscle hardness‐related genes of grass carp fed faba bean and found that the upregulated genes focused on the functional groups of myofibroblast proliferation, cytokine production, and nutrient metabolism. In the comparison of muscle transcriptomics of common grass carp and crisp grass carp, the collagen expression‐related genes were not identified among DEGs, whereas genes with muscle fiber differentiation‐promoting function, such as *MyoG* and *MyH9*, were significantly upregulated [42], which suggests that genes capable of promoting myofiber differentiation function, rather than the increase of collagen, play a more important role in improving muscle hardness. In this study, the myofiber density in the FB45 group was significantly increased, indicating that faba bean may promote the myofiber proliferation of shrimp. More research is required to investigate the regulating mechanism in the future.

## 5. Conclusion

In a practical diet containing 200 g/kg fish meal, the inclusion of 450 g/kg FB successfully substituted 225 g/kg soybean meal +225 g/kg wheat flour without adverse impacts on the growth performance of *L. vannamei*. Such substitution promoted the fresh water‐holding capacity, hardness, and myofibril density and increased the expression of myofibril‐related pathways and genes.

## Conflicts of Interest

The authors declare no conflicts of interest.

## Author Contributions

**Zhengri Gan:** methodology, formal analysis, investigation, data curation, writing – original draft preparation. **Yuting Xu and Xinyi Fei:** investigation, methodology. **X** **iaoqin Li:** supervision, project administration, writing – review and editing. **Xiangjun Leng:** funding acquisition, conceptualization, supervision, writing – review and editing. **Zhengri Gan** and **Yuting Xu** contributed equally to this work and should be listed as co‐first authors.

## Funding

This work was supported by the National Key Research and Development Program of China (Grant 2024YFD2402005).

## Data Availability

All data generated or analyzed during this study are included in this article.
